# Physical predictors of competitive success in Hungarian junior tennis: insights from a national talent identification program

**DOI:** 10.3389/fspor.2026.1819467

**Published:** 2026-06-24

**Authors:** Rita Géczi, Gergely Géczi, László Tóth

**Affiliations:** 1 School of Doctoral Studies, Hungarian University of Sports Science, Budapest, Hungary; 2 Sport Economics and Decision Making Research Center, Hungarian University of Sports Science, Budapest, Hungary; 3 Department of Psychology and Sports Psychology, Hungarian University of Sports Science, Budapest, Hungary; 4 Teacher Training Institute, Hungarian University of Sports Science, Budapest, Hungary; 5 Institute of Health Promotion and Sport Sciences, Faculty of Education and Psychology, Eötvös Loránd University, Budapest, Hungary

**Keywords:** agility, physical testing, power, speed, youth tennis

## Abstract

This study examined the predictive value of selected physical performance indicators in elite junior tennis players within Hungary's national development program. Forty athletes (23 boys, 17 girls), aged 11–18 years, participated in the 2024 standardized physical assessment conducted by the Hungarian Tennis Federation (HTF). Eleven motor performance tests were administered to evaluate key fitness domains, including speed, agility, coordination, explosive power, flexibility, reaction time, and core endurance. Based on preliminary correlation analyses and their theoretical relevance for tennis performance, four variables demonstrating the strongest association with ranking were selected for inclusion in the regression model: the 20-meter sprint, agility *T*-test, standing long jump, and plank hold. This approach allowed the construction of a parsimonious model while retaining variables widely recognized as relevant physical determinants of tennis performance. The regression model explained 49.2% of the variance in ranking outcomes (*R*^2^ = 0.492, *p* = 0.001). The 20-meter sprint time (*β* = 0.977, *p* < 0.001), agility *T*-test performance (*β* = −0.456, *p* = 0.013), and standing long jump distance (*β* = 0.538, *p* = 0.020) were significant predictors of national ranking, whereas plank hold duration did not show a statistically significant effect. These findings underscore the importance of sprint performance and lower-body explosive power in competitive success among junior tennis players. Although agility was significantly associated with ranking, its inverse direction suggests that pre-planned change-of-direction speed alone may not fully reflect the multifactorial determinants of competitive performance in youth tennis.

## Introduction

Tennis is a multifaceted sport that integrates technical, tactical, and physical components, all of which place high demands on athletic performance. One of the key determinants of competitive success is the development of conditional physical abilities, such as speed, agility, strength, endurance, and coordination—particularly in youth athletes, who possess substantial adaptive potential due to their stage of biological development. International sports science guidelines consistently emphasize the importance of targeted physical conditioning and its systematic monitoring through objective, standardized testing protocols.

Since 2019, the Hungarian Tennis Federation (HTF) has conducted annual physical assessments for elite junior tennis players aged 8–18. These evaluations follow a standardized protocol designed to assess key performance domains such as speed, explosive power, agility, endurance, core stability, and coordination. The longitudinal structure of this testing program enables not only the assessment of current physical status but also the tracking of individual developmental trajectories over time.

The aim of the present study is to examine the relationship between physical performance and competitive success among Hungarian junior tennis players, based on national testing data. By identifying predictive physical parameters, the findings aim to contribute to the refinement of evidence-based youth development models in tennis.

Competitive success in junior tennis is widely recognized as a multifactorial outcome influenced by technical, tactical, psychological, and physical components. Within this broader talent development context, physical performance indicators represent only one dimension of performance potential. However, objective physical testing can provide valuable insight into the athletic capacities that support match play demands, particularly in youth development systems where long-term monitoring of performance indicators is essential. Therefore, examining the association between standardized physical performance measures and national ranking may contribute to a better understanding of how physical attributes relate to competitive outcomes in junior tennis.

### International testing protocols and aerobic capacity in tennis

Leading international tennis organizations—including the International Tennis Federation (ITF), the Lawn Tennis Association (LTA), and the United States Tennis Association (USTA)—place a strong emphasis on the standardized and regular physical assessment of junior players. For instance, the ITF recommends the 20-meter shuttle run as a reliable indicator of aerobic capacity, as it simulates sport-specific movement patterns characteristic of tennis, such as accelerations, decelerations, and directional changes (27). According to the *ITF Coaching Review*, the primary goal of testing is to monitor physical development and to objectively evaluate speed, agility, and endurance (24).

The LTA's Pro Scholarship Programme incorporates shuttle test results as a criterion for national team selection, with benchmarks set at under 170 seconds for female and under 160 seconds for male athletes (28). Similarly, the USTA's fitness assessment protocol includes a variety of performance tests—such as the 20-yard sprint, push-ups, sit-ups, and vertical jump, among others—to provide a comprehensive evaluation of young athletes' physical capabilities (32).

These protocols share multiple parallels with the testing framework employed by the Hungarian Tennis Federation (HTF), which includes assessments of speed, agility, core stability, and upper-body explosive power. International benchmark values—such as shuttle test cut-offs and normative data for vertical jump performance—offer valuable reference points for calibrating national training and talent identification systems (22).

Given the intermittent nature of tennis, characterized by alternating low- and high-intensity efforts, aerobic capacity plays a critical role in sustaining performance throughout match play. Research indicates that over 75% of active playing time is spent at low intensity, whereas brief, high-intensity bursts between points are decisive for match outcomes (1, 20). Consequently, both aerobic and anaerobic energy systems are essential for optimal tennis performance (21, 30).

The Tennis Exhaustion Specific Test (TEST) provides a more precise assessment of sport-specific aerobic capacity, as it requires athletes to perform direction changes combined with stroke execution, thereby closely simulating real-match conditions (2, 3). Based on TEST results, significant performance differences have been identified between national- and international-level players, supporting the method's construct validity within tennis performance assessment (25, 26).

Speed and agility are widely recognized as critical performance determinants in junior tennis. Research indicates that these physical qualities are closely linked to chronological age, biological maturation, and sex-specific characteristics (4–6, 29). Longitudinal studies have confirmed that sprint and power performance can be effectively predicted by age, body composition, and lower-limb strength parameters (7).

The relevance of sport-specific movement patterns has been further emphasized by findings showing that holding a tennis racket during sprint testing significantly alters performance outcomes (8). The ability to rapidly change direction (COD speed) is especially critical in the U13–U15 age group, where developmental trajectories vary substantially (31). Targeted training can mitigate COD deficits and subsequently enhance on-court performance (6).

Additional research suggests that sprint training incorporating directional changes yields superior performance improvements in tennis players compared to linear sprint protocols (9). Holistic training models that simultaneously develop physical, technical, and mental skills have also been shown to significantly enhance speed-related performance (10).

Developing lower-body explosive power is essential for effective acceleration, directional changes, and stroke preparation. Multiple studies have demonstrated that 6–8 weeks of structured plyometric training can result in significant improvements in jump performance, COD ability, and serve efficiency in young tennis players (7, 23, 33). These gains are particularly pronounced among 12–13-year-olds, a developmental window characterized by rapid neuromuscular adaptation.

Both reactive and pre-planned agility can be enhanced through the integration of jump and sprint elements in training. According to Moya-Ramon et al. (11), resisted sprint training induces greater improvements in speed performance than conventional sprint drills, primarily due to its targeted development of the strength component essential for explosive actions.

Core muscle control is fundamental to maintaining functional movement patterns and preventing injuries—particularly during adolescence. Core training has been shown to improve dynamic balance, agility, and overall movement quality, as supported by outcomes from the Functional Movement Screen (FMS) (12–14). Given that low core stability increases the risk of musculoskeletal injuries, targeted core training is especially crucial for youth athletes (15).

Serve effectiveness and ball velocity are strongly influenced by upper-body strength, throwing-specific explosive power, and shoulder joint mobility. Research has revealed strong correlations between medicine ball throw performance, grip strength, and serve speed (16, 17). Shoulder mobility—particularly rotational range of motion—is also a key determinant of serve performance, particularly among male athletes (18).

The systematic development of upper-body strength and shoulder mobility has been shown to significantly enhance serve performance, as evidenced by objective measures of ball velocity and accuracy (19). Incorporating these training components into long-term athletic development programs is essential for achieving sustained success in competitive tennis.

The primary objective of the present study was to examine the relationship between conditional physical abilities and competitive success in elite junior tennis players, as reflected by their national ranking. Using standardized fitness test data collected during the 2024 autumn assessment period, the aim was to identify which physical parameters most strongly predict performance outcomes.

Although the current study is cross-sectional in nature—based on data collected at a single time point—it aligns with the longitudinal testing system developed by the Hungarian Tennis Federation. This integration provides a foundation for future time-series comparisons and individual performance tracking.

Our central hypothesis posited that sprint speed, agility, and lower-body explosive power would show significant associations with national ranking.

### Research question

To what extent are conditional physical abilities associated with the national ranking of junior tennis players?

## Methods

### Participants

A total of 40 elite junior tennis players participated in this study, all of whom were selected members of the Hungarian national junior team and took part in the official 2024 physical assessment organized by the Hungarian Tennis Federation (HTF). The sample included athletes from four age categories: U12 (9 boys, 8 girls), U14 (9 boys, 5 girls), U16 (4 boys, 2 girls), and U18 (1 boy, 2 girls), resulting in a total of 23 male and 17 female participants.

This study received ethical approval from the Research Ethics Committee (approval number: MTSE-KEB/No21/2025). All participants and parents/legal guardians provided written informed consent prior to participation.

All athletes held active competitive status at the time of testing, regularly participating in national and international tournaments and ranking events. Age data were calculated separately by age group and sex. Due to the small sample sizes in the U16 and U18 categories, these two groups were merged for statistical analysis in order to improve statistical stability and analytical feasibility. While this approach increased statistical feasibility, it may slightly limit the generalizability of the findings, as physical performance norms and developmental characteristics can differ between 16- and 18-year-old athletes.

Descriptive statistics on age distribution by group are presented in Table 1.

**Table 1:** Mean age (*M* ± SD) of participants by age group and sex.

Age group	Sex	*N*	Mean age (years)	SD
U12	Boys	9	11.91	0.56
U12	Girls	8	12.49	0.24
U14	Boys	9	13.91	0.67
U14	Girls	5	13.99	0.65
U16–U18	Boys	5	16.52	0.96
U16–U18	Girls	4	16.33	1.31

### Physical performance testing protocol

The data were collected during the 2024 national testing sessions following a standardized protocol established by the Hungarian Tennis Federation (HTF). The assessments aimed to evaluate key components of physical fitness, including linear speed, change-of-direction ability, coordination, muscular power, core endurance, flexibility, and reaction time.

A total of eleven validated motor performance tests were administered by three HTF-certified national coaches, all of whom had been trained in the federation's official procedures. Consistent verbal instructions and demonstrations were provided to ensure protocol standardization across participants.

To minimize fatigue, athletes rested for 1–2 min between trials and 3–5 min between different tests. All participants completed the tests in a fixed order, and for each test, the best performance was recorded.

The following tests were included in the battery:

- 20-meter sprint (linear speed)
- Agility test (change-of-direction ability)
- Hexagon test (coordination and reactive footwork)
- Standing long jump (lower-body explosive power)
- Five-direction sprint test (tennis-specific multidirectional speed)
- Sit and reach (flexibility)
- Reaction time test (response to visual stimuli)
- Medicine ball throw (upper-body power and tennis-specific coordination)
- Push-up test (upper-body endurance)
- Plank hold (core strength and endurance)

A detailed description of each test is provided in the subsequent section.

Competitive performance was defined based on the official national ranking published in October 2024. The aim of the statistical analysis was to determine the predictive value of selected physical performance variables in relation to national ranking outcomes.

### Description of physical tests

#### 20-Meter sprint

Assesses speed and acceleration. The test uses four electronic timing gates (two at the start line and two at the finish line) placed 20 m apart. Athletes start from a standing position behind the start line and sprint maximally upon the tester's signal. The best result from three trials is recorded. If timing gates are unavailable, a manual stopwatch can be used with visual start signals.

#### Agility test

Measures tennis-specific agility and change-of-direction speed. Cones are placed 4 m apart at the center of the baseline, with one tennis ball on each cone on one side. From a low ready position in the middle, the athlete moves laterally to transfer each ball from one side to the other, then returns to the starting position. Two trials are performed, and the best time is recorded.

#### Hexagon test

Evaluates coordination and reactive footwork. A hexagon is taped on the floor with each side measuring 0.5–0.6 m. Athletes jump in and out of the hexagon in a clockwise direction, following a specific pattern for three full rotations. Time is measured with a stopwatch. Touching the lines incurs a 0.5-second penalty; incorrect sequencing results in a 1-second penalty. The best of two attempts is recorded.

#### Standing long jump

Assesses lower-body power. Athletes jump forward from a standing position behind a marked line using both legs. The longest distance from the take-off line to the nearest heel contact is measured in centimeters. Multiple trials are allowed as long as improvement continues.

#### Five-direction sprint test

A tennis-specific multidirectional speed test. Five tennis balls are placed at predetermined court positions (e.g., corners of the baseline and T-junctions), and a racket is positioned at the center of the baseline. Starting behind the baseline center, the athlete sprints to retrieve the balls one by one and places them on the racket. The total time to complete all five retrievals is recorded. A 0.5-second penalty is added for each dropped ball. Two trials are allowed; the better result is kept.

#### Sit and reach

Measures flexibility of the hamstrings and lower back. Athletes sit barefoot with extended legs against a measurement box and reach forward with both hands overlapping. The distance beyond the toes is measured in centimeters, with the best of one trial recorded.

#### Reaction time test

Assesses response time to a visual stimulus. Athletes respond to a light cue by reacting as quickly as possible. The result is measured in milliseconds.

#### Medicine ball throw (forehand and backhand)

Assesses upper-body power and tennis-specific coordination. Using a 2 kg medicine ball, athletes perform two separate throws simulating forehand and backhand movements. Distance is measured in meters. The best result of each throw type is recorded.

#### Push-up test

Measures upper-body muscular endurance. Athletes perform as many technically correct push-ups as possible within 1 min. The total number of repetitions is recorded.

#### Plank hold

Assesses core strength and endurance. Athletes maintain a plank position for as long as possible with proper form. Duration is recorded in seconds.

### Data processing and statistical analysis

Data processing and statistical analyses were conducted using JASP, an open-source statistical software. A linear regression analysis was performed to examine the predictive value of physical performance variables. Assumption checks for normality and multicollinearity revealed no violations.

Selected physical performance variables were entered as independent variables, while national ranking served as the dependent variable. Although ranking represents an ordinal performance indicator, it was treated as a continuous variable in the present analysis to capture relative differences in competitive position among athletes and to enable exploratory modeling of associations with physical performance variables.

Based on preliminary correlation analyses and their theoretical relevance for tennis performance, four variables demonstrating the strongest association with ranking were selected for inclusion in the regression model: the 20-meter sprint, agility *T*-test, standing long jump, and plank hold. These variables represent key physical domains relevant to tennis performance, including acceleration speed, change-of-direction ability, lower-body explosive power, and core endurance.

Prior to model construction, descriptive statistics (mean ± SD) were calculated separately by sex for each test variable, as presented in Table 2.

**Table 2:** Descriptive statistics (mean ± SD) of physical performance tests by sex.

Test	Sex	*N*	Mean (*M*)	SD
Height (cm)	Boys	23	165.0217	13.87647
	Girls	17	163.4118	9.962798
Weight (kg)	Boys	23	53.43043	15.23433
	Girls	17	52.37647	11.32897
20 m sprint (s)	Boys	23	3.226957	0.218433
	Girls	17	3.38	0.1614
Agility *T*-test (s)	Boys	18	6.430556	0.423049
	Girls	13	6.423846	0.35359
Hexagon agility test (s)	Boys	18	12.24412	1.114215
	Girls	13	12.55538	1.236181
Standing Long Jump (cm)	Boys	23	196.8696	24.39319
	Girls	17	187.5294	17.62852
Five-direction sprint (s)	Boys	23	17.9113	1.204523
	Girls	17	18.33235	0.933632
Sit-and-reach test (cm)	Boys	23	6.869565	8.225815
	Girls	17	9.847059	9.286692
Reaction (s)	Boys	23	0.521783	0.086665
	Girls	17	0.523118	0.097298
Medicine ball forehand (m)	Boys	23	10.54783	2.512129
	Girls	17	9.044118	1.287304
Medicine ball backhand (m)	Boys	23	9.678261	2.036922
	Girls	17	8.861765	1.186319
Push up (reps)	Boys	23	26.65217	9.874709
	Girls	17	19.94118	6.368581
Plank hold (s)	Boys	23	180.4783	67.6185
	Girls	17	167.0588	54.13233
Shuttle run (shuttles)	Boys	14	90	21.93697
	Girls	9	78.75	13.98724

## Results

Gender differences were observed in several physical test results. On average, male athletes demonstrated faster 20-meter sprint times, greater upper-body strength (push-ups), and higher scores in explosive power tests such as the standing long jump and medicine ball throws. Female athletes, on the other hand, tended to score higher in flexibility (Sit-and-reach test) and core endurance (plank test), although these differences were not formally tested for statistical significance due to the sample size limitations. These patterns are consistent with developmental and physiological differences typically observed in adolescent athletes.

The aim of the linear regression analysis was to examine the extent to which selected physical performance variables could predict competitive success among junior tennis players, as measured by their official national ranking in October 2024. Four predictor variables were included in the model: 20-meter sprint time, agility *T*-test performance, standing long jump distance, and plank duration.

The coefficient of determination (*R*^2^ = 0.492) indicates that approximately 49% of the variance in national ranking can be explained by the model.

Based on the regression coefficients presented in Table 3, the 20-meter sprint, agility *T*-test, and standing long jump were found to be significant predictors of national ranking (*p* < 0.05). The results of the ANOVA further supported the model's overall fit (Table 4), indicating that the regression model was statistically significant [*F*_(4,26)_ = 6.29, *p* = 0.001]. These findings indicate that selected physical performance indicators are associated with national ranking outcomes in junior tennis players.

**Table 3:** Summary statistics of the linear regression model.

Model summary—ranglista (okt.ber)								
Model	*R*	*R* ^2^	Adjusted *R*^2^	RMSE	*R*^2^ Change	df1	df2	*p*
M_0_	0.000	0.000	0.000	3.385	0.000	0	30	
M_1_	0.701	0.492	0.414	2.592	0.492	4	26	0.001

M₁ includes 20 m sprint, agility, helyb.l t.vol, plank.

**Table 4:** Analysis of variance (ANOVA) evaluating the model fit.

ANOVA						
Model		Sum of squares	df	Mean square	*F*	*p*
M₁	Regression	169.034	4	42.259	6.291	0.001
	Residual	174.643	26	6.717		
	Total	343.677	30			

M₁ includes 20 m sprint, agility, helyb.l t.vol, plank. The intercept model is omitted, as no meaningful information can be shown.

The regression model was statistically significant [*F*_(4,26)_ = 6.29, *p* = 0.001], with the four physical performance variables collectively explaining 49.2% of the variance in national ranking (*R*^2^ = 0.492). The 20-meter sprint time showed the strongest predictive value (*β* = 0.977, *p* < 0.001), where a lower time indicates better performance. The standing long jump showed a significant predictive value (*β* = 0.538, *p* = 0.020), as longer jump distances reflect higher lower-body power. Agility performance was significantly associated with national ranking (*β* = −0.456, *p* = 0.013). The negative regression coefficient indicates that players with better ranking positions (lower ranking numbers) demonstrated longer agility completion times. In other words, higher-ranked athletes in this sample showed slightly slower performance in the agility test. The plank variable, where longer hold times indicate greater core endurance, did not show a statistically significant predictive value (*β* = 0.202, *p* = 0.169). While its role in predicting ranking is limited, previous research suggests its practical relevance for core strength and injury prevention, particularly in adolescent athletes.

Based on the standardized regression coefficients, sprint time, agility, and explosive lower-body power were significant predictors of ranking performance in junior tennis players.

Table 5 summarizes the standardized regression coefficients, along with their respective confidence intervals, providing a clear overview of the individual contributions of each physical performance metric to national ranking.

**Table 5:** Predictive value of physical performance tests for national ranking, standardized regression coefficients.

Coefficients								
Model		Unstandardized	Standard error	Standardized	*t*	*p*	95% CI	
							Lower	Upper
M₀	(Intercept)	5.452	0.608		8.968	<.001	4.210	6.693
M₁	(Intercept)	−63.812	23.422		−2.724	0.011	−111.956	−15.668
	20 m sprint	21.607	5.067	0.977	4.264	<.001	11.192	32.023
	Agility	−3.970	1.487	−0.456	−2.670	0.013	−7.027	−0.913
	Helyb.l t.vol	0.110	0.045	0.538	2.469	0.020	0.018	0.202
	Plank	0.010	0.007	0.202	1.413	0.169	−0.005	0.024

Collinearity diagnostics indicated elevated Condition Index values (approximately 136; Table 6), suggesting potential redundancy among some predictors, particularly between sprint and plank variables. However, as variance inflation factors remained within acceptable ranges, multicollinearity was not considered to substantially bias the regression estimates.

**Table 6:** Presents the detailed collinearity statistics, supporting the assumption that multicollinearity did not significantly distort the regression coefficients.

Collinearity diagnostics								
Model	Dimension	Eigenvalue	Condition index	Variance proportions				
				(Intercept)	20 m sprint	Agility	Helyb.l t.vol	Plank
M₁	1	4.884	1.000	0.000	0.000	0.000	0.000	0.005
	2	0.104	6.857	0.000	0.000	0.001	0.001	0.942
	3	0.010	21.973	0.000	0.011	0.039	0.212	0.004
	4	0.001	58.845	0.030	0.164	0.958	0.025	0.046
	5	2.629 × 10^−4^	136.305	0.970	0.824	0.002	0.761	0.003

The intercept model is omitted, as no meaningful information can be shown.

Based on the quantitative results, it can be concluded that sprint speed, change-of-direction ability, and lower-body explosive power significantly contribute to competitive performance in junior tennis. The objective values obtained through standardized physical testing may therefore serve as useful predictors of athletic success in this population.

This study identified sprint speed and lower-limb explosive power as consistent positive predictors of national ranking. Although agility performance showed a statistically significant association, its direction differed from theoretical expectations, suggesting that pre-planned change-of-direction speed alone may not fully explain competitive success in junior tennis. The prominent role of sprint time highlights the critical importance of acceleration during match play, particularly in scenarios requiring short reaction times and rapid positioning. These results are broadly consistent with existing international literature (e.g., 6, 7).

Agility *T*-test and standing long jump performance also showed significant associations with national ranking, underscoring the contribution of lower-limb strength and change-of-direction ability to overall performance. These factors may be especially influential in the U12–U14 age categories, where neuromuscular development is rapid and technical-tactical skills are still being refined.

Interestingly, the direction of the association between agility and ranking was contrary to theoretical expectations. Higher-ranked athletes demonstrated slower agility test times. Several factors may explain this unexpected finding. First, national ranking reflects competitive success that is strongly influenced by technical-tactical skills, perceptual abilities, and decision-making efficiency, which may compensate for minor differences in pre-planned change-of-direction speed. Second, the applied agility protocol primarily assessed pre-planned movement rather than reactive agility, which is more representative of match play. Finally, developmental heterogeneity within the sample (U12–U18) may have influenced the relationship between physical performance and ranking outcomes. This finding may also reflect the limitations of pre-planned agility tests in representing the perceptual and reactive demands of tennis-specific movement patterns during match play.

Although the plank variable did not reach statistical significance (*β* = 0.202, *p* = 0.169), it was retained in the model based on prior literature highlighting its relevance for core stability. However, its predictive role in relation to national ranking appears to be limited in this sample.

A key limitation of this study is the relatively small sample size, especially in the older age groups. Another limitation relates to the relatively wide age range of the sample (U12–U18). This reflects the structure of the Hungarian national development system, where the official national team typically consists of four players per sex and age category. Consequently, the available sample size within each group is limited. In addition, older junior players often compete in international tournaments during the testing period, which may further reduce participation in the national physical assessment sessions.

Furthermore, national ranking only partially reflects overall athletic performance, as technical, tactical, and psychological factors also play a substantial role. Ranking may also be influenced by contextual factors not controlled for in this study, including tournament participation frequency, strength of competition, and injury status. Nevertheless, the fact that nearly half of the variance in ranking (*R*^2^ = 0.492) could be explained by physical test results reinforces the important role of physical conditioning in junior tennis development.

## Conclusions

This study identified sprint speed, change-of-direction ability, and lower-limb explosive power as significant predictors of national ranking among junior tennis players. These attributes are not only key indicators of general fitness status but also reflect match-specific demands, such as rapid acceleration and positioning.

The standardized testing protocol applied by the Hungarian Tennis Federation aligns well with international guidelines and provides a robust foundation for systematic athlete evaluation. By integrating such assessments into long-term development pathways, training programs can be better tailored to individual needs, especially during periods of accelerated neuromuscular adaptation.

Nevertheless, several limitations must be considered. The study's cross-sectional design precludes causal inference and limits interpretation to associations between physical performance indicators and competitive ranking. Furthermore, performance differences linked to biological maturity and sex could not be formally assessed, as no indicators of maturation (e.g., PHV, Tanner stage) were collected. In addition, the inverse association observed for agility should be interpreted with caution, as developmental heterogeneity across age categories and the use of a pre-planned change-of-direction protocol—rather than a reactive, tennis-specific assessment—may have influenced the direction and magnitude of the observed effect. Future research should incorporate maturity indicators and adopt longitudinal or repeated-measure designs to track developmental changes over time.

Finally, while physical performance is clearly influential, competitive success in tennis is multifactorial. Incorporating technical, tactical, and psychological assessments would offer a more holistic understanding of talent development. The present findings contribute to evidence-based practice in youth tennis and support the refinement of national training curricula and selection policies.

### Practical applications and recommendations for junior tennis development

Based on the findings of this study, the following practical recommendations are proposed for the physical development programs of junior tennis players in Hungary:

1. Prioritizing Speed Development: Given the strong predictive value of 20-meter sprint time, speed training should be a central focus in program design. Incorporating exercises that target short-distance acceleration and reaction time may contribute to improved competitive performance.
2. Targeted Agility and Change-of-Direction Training: Due to the critical role of change-of-direction (COD) speed, both reactive and pre-planned agility should be systematically developed. Regular inclusion of COD-specific drills during preparatory periods is recommended.
3. Enhancement of Explosive Power and Lower-Limb Strength: The significant relationship between standing long jump and performance outcomes supports the integration of plyometric training and lower-body strength programs. This is particularly important in the 12–14 age group, where neuromuscular adaptation is most responsive.
4. Core Stability Development for Injury Prevention: Although the plank test was not a significant predictor in the regression model, core muscle strength remains essential for executing technically sound movement patterns and reducing injury risk. Both static and dynamic core training should be implemented across all age categories.
5. Continuation of Standardized Testing and Data Collection: Maintaining a unified system of physical assessments and building a long-term athlete monitoring database will allow for individualized tracking of physical development. Integrating test results into personalized training plans can support targeted and efficient athletic development.
6. Application of an Interdisciplinary Approach: Given the multifactorial nature of competitive success, it is advisable to incorporate objective assessments of technical, tactical, and psychological components alongside physical testing. This integrated approach enables the creation of a holistic athlete profile.

In addition to the above, the standardized monitoring of key physical attributes on an annual basis is strongly recommended. This enables the adaptation of training programs to individual developmental trajectories. These findings reinforce the central role of physical fitness in junior tennis, not only as a foundation for technical and tactical execution but also as a standalone predictor of competitive success.

## Data Availability

The raw data supporting the conclusions of this article will be made available by the authors, without undue reservation.
